# Obtaining person-related information from employees with chronic health problems: a focus group study

**DOI:** 10.1007/s00420-019-01440-5

**Published:** 2019-05-18

**Authors:** Mariska de Wit, Haije Wind, Carel T. J. Hulshof, Angela G. E. M. de Boer

**Affiliations:** Coronel Institute of Occupational Health, Amsterdam Public Health Research Institute, Amsterdam UMC, University of Amsterdam, PO Box 22700, 1100 DE Amsterdam, The Netherlands

**Keywords:** Occupational physicians, Insurance physicians, Person-related factors, Work participation, Focus group

## Abstract

**Purpose:**

The objective of this focus group study is to assess how occupational physicians (OPs) and insurance physicians (IPs) can best obtain information concerning person-related factors from employees. The research question was: what is the most effective way for OPs and IPs to obtain information concerning person-related factors, in the opinion of employees with chronic health problems?

**Methods:**

Three focus group discussions were conducted comprising of a total of 23 employees with work limitations due to chronic health problems. Employees discussed how physicians could best obtain information related to ten person-related cognitions and perceptions that are associated with work participation. The discussions were recorded, transcribed verbatim and analyzed through qualitative content analysis.

**Results:**

Employees indicated that information addressing person-related factors could best be obtained through discussing them directly during consultations, as opposed to the use of questionnaires or diaries. Important prerequisites to having fruitful conversations include a mutual trust between employee and physician, a sense of genuine physician interest, and the understanding of the physician of employees and their health concerns. Employees described various factors that influence these conversations, including the knowledge and communication skills of physicians, employee anxiety, and the atmosphere and time frame of the consultation.

**Conclusions:**

Information concerning the person-related factors of employees can best be obtained by discussing them during consultations. However, there has to be mutual trust, interest and understanding before employees feel comfortable to talk about these factors with a physician. OPs and IPs should consider these, and other identified factors, when asking about person-related factors during consultations.

## Introduction

Having a chronic disease can negatively impact participation in work (De Boer et al. 2018; Jetha et al. 2017). Occupational physicians (OPs) and insurance physicians (IPs) can play an important role in increasing work participation and limiting sickness absence under employees with a chronic disease, by intervening on factors which influence work participation (Dekkers-Sánchez et al. 2013; Verbeek 2006). Certain perceptions and cognitions—such as motivation, self-efficacy, and expectations regarding recovery or return to work (RTW)—are important person-related factors that influence work participation (Hallegraeff et al. 2012; Boyle et al. 2014; Besen et al. 2015). A systematic review by De Wit et al. (2018) demonstrated an association between work participation and ten person-related factors: expectations regarding recovery or RTW, optimism/pessimism, self-efficacy, motivation, feelings of control, perceived health, coping strategies, fear-avoidance beliefs, perceived work-relatedness and catastrophizing. For example, catastrophizing and fear-avoidance beliefs were associated with an increased time until RTW, whereas having positive expectations concerning RTW or recovery was a predictor of a shorter time until RTW (De Wit et al. 2018). Previous qualitative research has shown that both employees and physicians view person-related factors as important in work participation (Dekkers-Sánchez et al. 2013; Matérne et al. 2017; Van Muijen et al. 2015; Dunn et al. 2010; Wilbanks and Ivankova 2015), making these factors key targets for interventions to increase work participation.

To intervene effectively on these factors, it is imperative that OPs and IPs are able to obtain information concerning those person-related factors that encourage or hinder work participation in employees. This can be achieved through physician–patient interaction during consultations. However, to obtain information concerning these factors, it is crucial that employees disclose information about these factors. Physician use of specific communication skills, such as asking open-ended questions and active listening, can encourage patients to share information about themselves (Ha and Longnecker 2010; Lewis et al. 2011; Matusitz and Spear 2014). It is possible that these techniques may also encourage employees to disclose more information concerning person-related factors during consultations.

This is, however, dependent on the communication skills of the individual physician. Physicians and patients can differ in their interpretation of physician communication skills; physicians who think they are communicating well may not always be perceived as good communicators by their patients (Kenny et al. 2010). These discrepancies can further limit the disclosure of important patient information, such as that concerning person-related factors. To enhance physician–patient communication and facilitate the disclosure of information regarding relevant person-related factors, it is important to evaluate patients’ opinions concerning how these factors should be discussed. The opinion of employees regarding how physicians should obtain person-related information is, however, yet unstudied. This study, therefore, poses the research question: what is the most effective way for OPs and IPs to obtain information concerning person-related factors, in the opinion of employees with chronic health problems?

## Method

This qualitative study utilizes three focus group discussions (FGDs). We chose this study method because FGDs allow for the collation of a diverse range of participants and opinions: for example, through the inclusion of employees with different disabilities and different experiences with OPs and IPs. The moderator of a FGD can respond to questions from participants about complex or academic subjects (e.g. person-related factors) and can request more detailed responses from participants when clarification of their responses is needed (Wong 2008). The consolidated criteria for reporting qualitative research (COREQ) were used to comprehensively report the focus group process (Tong et al. 2007).

### Participants

FGD participants were recruited via a panel of more than 23,500 patients from the Patient Federation in the Netherlands, an association representing 170 patient and consumer organisations. In February 2018, members of the panel were invited by email to participate in one of the focus groups. In addition, four consumer organisations affiliated with the Patient Federation (Lung Foundation Netherlands, Heart Council, Kidney Patients Association Netherlands and Care Importance Brabant) were approached and agreed to send invitations to their members. Individuals were eligible to participate if they were employees who had experienced limitations during paid work due to chronic health problems, spoke Dutch fluently and were between 18 and 67 years of age. Employees who expressed interest in participating received information by email detailing the purpose of the FGDs, the person-related factors that would be discussed, the professional background of the interviewers, and possible dates for the FGDs.

Thirty employees agreed to participate in the study. Participants were assigned to one of the three focus groups, with the aim of achieving an equal spread of gender, age and disabilities over the groups. Three of the 30 employees who agreed to take part in the study were unable to participate due to other appointments or due to health problems. Four employees did not attend for reasons unknown. In total, 23 employees participated in the study, divided between the three focus groups (focus group A and B both had seven employees, and focus group C consisted of nine employees). Demographics of the participants are presented in Table 1.

**Table 1:** Demographic variables (gender, age, disability)

	Focus group A, *n*/*N*	Focus group B, *n*/*N*	Focus group C, *n*/*N*	Total, *n*/*N*
Gender				
Male	3/7	4/7	4/9	11/23
Female	4/7	3/7	5/9	12/23
Age, mean (SD)	57.0 (5.7)	57.1 (4.6)	51.1 (8.2)	54.7 (7.1)
Disability				
Physical disability	6/7	4/7	4/9	14/23
Mental disability	–	2/7	4/9	6/23
Physical and mental disability	1/7	1/7	1/9	3/23

### Procedure

The three FGDs were conducted between March and April 2018 at the Amsterdam UMC, location Academic Medical Center in Amsterdam. The moderator for each FGD was one of two male authors (CH or HW), respectively OP and IP. Both are employed at the Coronel Institute of Occupational Health, have a Doctorate of Medicine and of Philosophy and have previous experience in qualitative research and conducting FGDs. The discussions were recorded with an audio recorder, and field notes were taken by another author (MdW). The authors did not know the participants before the FGDs. Apart from the researchers and participants, no-one else was present during the FGDs.

Before the start of each 2-h FGD—all of which were conducted in Dutch—each participant signed an informed consent form. The FGDs started with an explanation of the purpose of the discussion, a brief introduction of the participants and an explanation of the structure of the FGD by the moderator. During the discussion that followed, the primary question addressed was: what is the most effective way for OPs and IPs to obtain information concerning person-related factors? The person-related factors defined were ten factors identified in a preceding systematic review (De Wit et al. 2018). The person-related factors were explained through ten case descriptions, presenting fictional situations in which the factor in question influenced the work participation of an employee with chronic health problems. During the discussion, the participants were encouraged to speak openly about their views and thoughts. When needed, the moderator asked the participants to clarify their answers. At the end of each FGD, participants received a travel allowance and a gift card of 25 euros in return for their participation.

### Data analysis

The recordings of the discussions were transcribed verbatim and anonymized. We did not send the transcripts back to the participants for comments or correction, and we did not ask for feedback on the findings. For data analysis purposes, we used qualitative content analysis (Mayring 2014). The transcripts from the FGDs were coded using MAXQDA 12 Software (VERBI Software 2017). Codes were assigned by one author (MdW) to segments of the transcript of the first two FGDs. These were then checked by a second author (HW). Disagreements about the coding were resolved by discussion. A coding framework consisting of main themes and subthemes was built by categorizing the codes. The main themes and subthemes were discussed between all authors until a consensus about the framework was reached. Following author consensus regarding the codes and coding framework, the transcript of the third FGD was coded using the coding framework by one author (MdW). The different themes of the coding framework are described in the “Results” section. To illustrate our findings, we have included quotations of participant discussions from the focus groups. A native English speaker translated these from Dutch into English.

## Results

### Coding framework

Four primary themes of discussion were identified from the FGD transcripts. They were defined as the main categories for the coding framework: (1) methods to obtain information concerning person-related factors, (2) prerequisites for talking about person-related factors during consultations, (3) positive influences on conversations concerning person-related factors, and (4) negative influences on conversations concerning person-related factors.

### Methods to obtain information concerning person-related factors

Participants largely acknowledged the importance of obtaining person-related information and talked about three different ways to do this. In Table 2, the methods identified with the corresponding quotations of participants are presented.

**Table 2:** Identified methods to obtain person-related information

Method	Citation examples
Diary	Participant B3: “When you have those invisible consequences and, as a doctor, you want to find out: what is it? Fellow sufferers I know have sometimes compiled a weekly schedule. Every half hour. With a lot of gaps. Then the doctor asks: what are the gaps? They are the rest breaks I need. This could help you to find out what the weekly schedule of that man or woman is roughly like. And draw conclusions from that.”
Questionnaire	Participant A6: “A checklist is also always dangerous, because it only lists the answers that you have never thought of before, but you never have room, or often don’t have room, to write down what you are experiencing or what you have not thought of.” Participant A5: “(…) And who reads it? I’m not going to write everything down if I don’t know who will read it.”
Discussing factors within consultations	Participant A5: “(…) So if I have good contact with someone and feel that I’m able to speak out, that also gives you a sense of security.”

One method for the physician to obtain information, according to the FGD participants, is to ask the employee to keep a diary and to discuss this during consultations. Employees may thereby record information such as their activities or feelings. In the opinion of some of the participants, discussing this diary with employees can help physicians to gain insight into the limitations the patient faces during the day and into the patient’s cognitions and perceptions around this.

A second method described by participants was the use of a checklist or questionnaire. But participants expressed skepticism about using this method. They voiced concern that using a standardised preformat or checklist may limit the comprehensiveness of the answers an employee provides. Some participants felt that employees may not always give honest answers due to a fear that other people than the physician may read their answers.

Partially due to these limitations of checklists and questionnaires, most employees preferred to discuss the factors directly during their consultations with the physician. In contrast to keeping a diary and completing questionnaires, all participants had experience with consultations; this method, therefore, provided the bulk of discussion during the FGDs. Different factors were identified that could influence the effectiveness and development of conversations pertaining to person-related factors.

### Prerequisites for obtaining information during consultations

Before effective questions can be asked by physicians about person-related factors during consultations, FGD participants defined a set of prerequisites they felt to be of importance. Table 3 shows these identified prerequisites, with corresponding quotations from the participants.

**Table 3:** Prerequisites for discussing person-related factors during consultations

Prerequisites	Citation examples
Mutual trust between employee and physician	Participant B5: “(…) I agree with you: there needs to be an element of trust in the first instance and only then you can engage in discussion. Otherwise you can’t.” Participant C5: “So when it comes to the point where you are discussing personal factors, things really close and personal, then there needs to be a bond of trust.”
Showing interest and involvement	Participants B4 and B2: “You want to be seen as a human being and not…” “…Just as a number.”
Understanding	Participant C5: “(…) And that he acknowledges that you have those fears. That it’s normal and that you can talk about it. I think that really helps a lot.”

The most important prerequisite was a mutual trust between the employee and the physician. Trust is an important factor that can facilitate the disclosure of information. All participants agreed that without this trust, a meaningful conversation about person-related factors was not possible.

A second prerequisite was that the physician shows interest or demonstrates involvement with the employee. Participants agreed that it is important that employees feel they are being heard by the physician, and that, subsequently, obtaining information about person-related factors would be facilitated during the conversation when the physician shows a genuine interest in their situation and makes the employees feel like an individual.

The last described prerequisite was the understanding of the physician. Participants felt that it was important that the physician understands the employee’s feelings and cognitions and acknowledges that these are not unusual.

### Positive influences on the development of conversations concerning person-related factors

Over the FGDs, it became apparent that a number of factors can positively influence the instigation and development of a conversation about person-related factors. These factors can be broadly divided into three different subthemes: (1) communication skills of the physician, (2) context of the conversation, and (3) knowledge of the physician, and are detailed in Table 4 along with corresponding quotations from participants.

**Table 4:** Positive influences on the development of conversations concerning person-related factors

Positive influences	Citation examples
**Communication skills of the physician**	
Listening	Participant B1: “That people judge instead of remaining open and listening, because if they listen to you they’ll soon hear that you would very much like to go to work.”
Asking open questions	Participant B6: “Don’t ask closed questions.”
Explaining what is realistic and defining boundaries	Participant A5: “(…) I think it’s good if the occupational physician makes an effort to.. yes, generate some kind of awareness in someone. About what is genuinely realistic.”
Focusing on getting better instead of returning to work	Participant A2: “(…) The patient’s first priority is recovery. And… I think that that should also be something that the occupational physician focuses on. The first priority is to get better or if you can’t get better to learn to deal with the situation you’re in.”
Coaching and offering help	Participant A5: “I don’t need to hand over control, I consider it my responsibility, but coach me, I’m very willing.”
Setting small goals	Participant C6: “If the occupational physician maybe looks at his home situation, what he’s doing at that moment and then sets small targets to see what progress can be made and what problems he faces. Then you can also see, yes, whether there is progress and whether he can take on certain things. And also where his problems lie, what’s going wrong.”
Expressing appreciation	Participant B5: “(…) But it’s important to keep hearing that you’re on the right track. That’s good.”
**Context of the conversation**	
Taking enough time	Participant A3: “Particularly here I think, that’s why I feel that it’s so important to invest time at the start, because you don’t usually discuss it in the first meeting but if you actually invested time in the first meeting, it might be easier to broach in the third of fourth meeting (…)”
Atmosphere of the conversation	Participant C1: “But the first thought that came to mind was: it really makes a difference what atmosphere you are entering.”
**Knowledge of the physician**	
Having knowledge about the employee	Participant B5: “The better you know the person sitting opposite you, and that it’s great if you know who is sitting opposite you. What are your hobbies? Because if you can’t work, but you do walk to your vegetable patch every day, so to speak. It must be possible to make some kind of link and then you can connect it back to your work.”
Having knowledge about problems/complaints of the employee	Participant B3: “(…) Try to get to the bottom of what that person is really suffering from.”
Having knowledge about the working environment of the employee	Participant A3: “I think it may be easier to engage in discussion with an occupational physician if they make it clear that they understand the company and your working environment.”

#### Communication skills of the physician

Participants viewed it as very important that physicians listened carefully to employee responses, to prevent misinterpreting information about certain person-related factors. Furthermore, physicians should avoid closed questions and ask open questions to facilitate discussion around person-related factors during consultations. Such open questions may be focused on a variety of topics. Important themes to ask about included the work of the employee (e.g. “What adaptations have already been made?”), the employee’s private situation (e.g. “What do you do on a day?”), the future of the employee (e.g. “How do you think you will continue in the future?”), the employee’s complaints or concerns and what had been done to address them (e.g. “What are you struggling with?” and “What process have you started to recover?”) and how the physician could help the employee (e.g. “What do you need to be able to resume part of your work?”). Some participants felt that it was important to end the conversation with a question about how the employee experienced the current consultation with the physician (e.g. “How did you find this consultation?”), in order for the physician to be able to improve future conversations concerning person-related factors with employees.

It is crucial that the physician makes the employee aware of what improvements are realistic and defines boundaries for the activities of the employee. The physician should focus on regaining health rather than returning to work. The consultation was felt to run more smoothly when the physician adopted the role of a coach. The physician should give tips for the employee to improve their current situation, should set small goals for the employee and should show appreciation when small goals are reached, or progress is made.

#### Context of the conversation

FGD participants emphasized the value of leaving enough time in consultations to discuss person-related factors and structuring successive consultations accordingly. Some participants felt that physicians should not address these factors immediately but should wait until sufficient rapport is established between physician and employee to allow the employee to feel comfortable to discuss them. Some employees even thought that a physician should not begin to address the factors until the second or third consultation. It is essential that the overall atmosphere of the conversation is pleasant before the physician starts to talk about the factors.

#### Knowledge of the physician

Participants agreed that a physician would obtain more information about person-related factors if they developed greater personal knowledge of the employee. Physicians need to be aware of the intellectual level of the employee, therefore, they can adapt their way of talking accordingly. Also paramount was that the physician had sufficient information about the disease or disorder of the employee and the (invisible) impairments that might exist as a result of this. The physician needs to be aware that the employee complaints and corresponding cognitions and perceptions may differ between individuals and can change over time. In addition to this, discussions around person-related factors were described to be more effective when the physician knew something of the company, the employer and the corporate culture in which the employee works.

### Negative influences on the development of conversations concerning person-related factors

Aside from positive factors, participants also discussed issues that negatively influenced the instigation and development of a conversation. These negative influences described were diverse, but can be broadly divided into four different subthemes: (1) negative influences of the occupational health and social security systems, (2) negative influences of the physician, (3) negative influences of the employee, and (4) negative influences of the employer. Table 5 summarises the different negative influences and provides some corresponding quotations from participants.

**Table 5:** Negative influences on the development of conversations concerning person-related factors

Negative influences	Citation examples
**Negative influences of the occupational health and social security systems**	
Physician not being accessible	Participant C3: “Here, things are arranged in such a way that you’re obliged to make an appointment with the occupational physician via the consultant. Otherwise, you just don’t have access.”
Lack of contact with physicians	Participant C2: “(…) After six months or a year, I was still ill and then had completely different occupational physicians again, and I didn’t have to go to the labor expert anymore because they said the situation was clear. And then suddenly I don’t hear anything anymore.”
Employees being allocated different physicians	Participant C2: “(…) That’s right, because I never spoke to the same doctor again throughout the entire process. (…). I’m always dealing with different people, so I, I just don’t know them.”
Focus on money	Participant C1: “Putting the employee first—I have the feeling that it is more about putting costs first.”
Not taking into account the reduced income of the employee	Participant C8: “Everyday aspects of life are often forgotten. That you have a loss of income and a family to support and have to get by on 70% and it often gets forgotten what all that involves (…)”
Employees not receiving adequate information about the process	Participant C1: “I have no idea who I’m going to speak to or when.”
**Negative influences of the physician**	
Lack of physician time	Participant C2: “(…) I don’t know if they’ll manage it in the time that he has.”
Not asking about person-related factors	Participant C1: “Some questions aren’t even asked by the occupational physician.”
Exerting too much pressure to return to work	Participant A2: “(…) Yes, all that guy ever does is try to get me back to work as soon as possible… I say nothing, because he may actually be able to find a gap that (…)”
**Negative influences of the employee**	
Anxiety in general	Participant A7: “(…) I do feel anxious in one-to-one discussions with the occupational physician.”
Anxiety about disability assessment	Participant C2: “(…) But now I find I’m bracing myself for the UWV (Employee Insurance Agency) doctor who will assess me.”
Anxiety about disclosing information	Participant B7: “I’m not honest about that. I pretend there’s nothing wrong with me.”
**Negative influences of the employer**	
Communication/cooperation between employer and physician	Participant C1: “And I think that an occupational physician if he would have an independent position, and not be paid by the employer or the UWV. But genuinely independent, just like a general practitioner.”
Conflicts between employer and employee	Participant C2: “(…) And before that I had a job with a manager who was an absolute monster. I would have preferred to have reported sick back then, something along the lines of: I’ve got you, than at the place where I was working at the time I reported sick.”

#### Negative influences of occupational health and social security systems

A significant negative influence on conversations described by participants was a low frequency of contact between employees and physicians. Physicians were often not accessible and getting in touch with them could prove very difficult. FGD participants sometimes did not have any direct contact with OPs, and only had contact with a designated case manager. This makes discussing person-related factors with OPs impossible. In contrast, other participants stated that discussions around person-related factors could be impeded by continually changing the physician they had contact with, and so, despite multiple consultations, they would never see the same physician twice.

Another factor described as negatively influencing employee–physician conversations was that participants felt that social security organisations and employers were often focused on financial issues, rather than the wellbeing of employees. Participants stated that sometimes economic interests would seem to be more important than human interests. Other participants felt that the physician’s role was merely to limit the costs of the employer, instead of helping employees to get better. Despite this perceived overemphasis regarding money, many participants felt that physicians did not always take the reduced income of the employee into account. Feelings such as this lead to distrust towards the physician and this can disrupt and impede conversations about person-related factors.

A final negative influence of the occupational health and social security systems is that employees often have little knowledge of the working practices of OPs and IPs, and about the disability assessment. Participants described that it is not always clear when they need to talk to physicians and where employees should go to get more information regarding this. This lack of adequate information can lead to uncertainty and anxiety in employees, which in turn can have negative consequences in developing conversations concerning person-related factors.

#### Negative influences of the physician

Participants also described that the physician could exert a negative influence on conversations pertaining to person-related factors. A lack of time on the part of the physician—specifically not taking the time to ask about person-related factors—will limit the possibility of obtaining person-related information. Some participants felt that physicians sometimes put too much pressure on employees to return to work, which may, in turn, have a negative influence on the development of the conversation.

#### Negative influences of the employee

Almost all FGD participants agreed that employee feelings of anxiety could negatively impact conversations concerning person-related factors. Most of this anxiety appeared to be centered around the disability assessment by the IPs, with employees reticent to disclose too much information for fear of negative consequences for the disability assessment. Other participants described anxiety around disclosing too much or too little information towards colleagues and employers concerning their health problems.

#### Negative influences of the employer

The employer can also have a negative impact on the conversation between employee and physician. Owing to the communication between the employer and physician, FGD participants felt that the confidentiality usually afforded to doctor-patient interactions was not present, leading employees to lack the feeling of trust needed to open up in conversations. These feelings of distrust can be increased when there are conflicts between the employee and employer.

## Discussion

### Key findings

Employees with work limitations due to chronic health problems acknowledge the importance of person-related factors in their management and are most comfortable sharing these factors with OPs and IPs directly in consultations. Trust, understanding and interest were considered essential to allow effective discussion or conversations concerning person-related factors. Aside from these prerequisites, issues pertaining to the communication skills of the physician, the knowledge of the physician, and the context of the consultation were identified being able to impact the development of the conversation positively. Employees identified issues related to occupational health and social security systems, the physician, the employer and the employee which can negatively influence the instigation and development of such conversations.

Trust between employee and physician was perceived as the most important prerequisite for obtaining person-related information during consultations. This is in accordance with previous studies that describe the importance of trust for patients in disclosing information during conversations about medical issues (Julliard et al. 2008; Main et al. 2010; Kelak et al. 2018). An interview study by Julliard et al. (2008) identifies trust, compassion and respect, as prerequisites for patients sharing health information with their physician. Studies by Main et al. (2010) and Kelak et al. (2018) also emphasize the importance of trust for disclosing information during consultations.

According to Ridd et al. (2009) and Skirbekk et al. (2011), trust arises when patients and physicians spend more time with each other in consultations. This is consistent with our findings that employees valued physicians taking time to develop a mutual trust before addressing person-related factors. This association between spent time in consultations and trust could also help to explain why a lack of contact with the physician and limited accessibility were perceived as negative influences on the development of conversations concerning person-related information. In addition, employees described the negative influence of seeing different physicians each time. All of these factors limit the time that employees spend with the same physician, potentially disrupting the process of building trust (Ridd et al. 2009; Skirbekk et al. 2011). Appropriate timing of conversations about person-related factors—as well as taking enough time to discuss them—are essential for obtaining reliable person-related information during consultations.

Other prerequisites for obtaining information about person-related factors involved the physician showing interest, being involved and understanding. This is consistent with results of a review by Ridd et al. (2009) showing that patients value doctors who appear interested during consultations, and results of a study by Kelak et al. (2018) in which involvement of the physician was identified as a critical component for patients to disclose information. The results are also supported by a study by Mazzi et al. (2016), in which taking the patient seriously and treating the patient as a person were identified as two of the five most important recommendations from patients for physicians to make consultations more effective.

Participants of the FGDs identified, in addition, a number of different factors that may influence the development of the conversation about person-related factors. Several factors, such as listening, asking open questions, and having knowledge about the patient’s complaints have also been identified in other studies as important factors for the development of medical consultations (Main et al. 2010; Ha and Longnecker 2010; Julliard et al. 2008; Ranjan et al. 2015; Mazzi et al. 2016; Kelak et al. 2018). Other studies also identified factors which were important for the development of the consultation, that were not mentioned by our participants, such as the importance of non-verbal signals from physicians, like keeping eye contact with the patient (Main et al. 2010; Bensing et al. 2011; Deledda et al. 2013).

### Strengths and limitations

A strength of our study is that the focus groups consisted of participants with different types of disabilities, making the findings generalizable to employees with various health problems. Another strength is that the experiences of the patients with physicians diverged from positive to very negative, providing information about both facilitators and barriers to obtaining information about person-related factors.

A limitation of this study is that participants had difficulty answering some of the questions asked during the FGDs. Instead of talking about how to obtain information about cognitions and perceptions, participants had the tendency to talk about different ways to change the cognitions and perceptions of the employee. Although this information can be useful in future research, it was not included in this study because it did not help us in answering our research question.

### Implications for practice and future research

We recommend that physicians consider person-related factors during their consultations to increase work participation in employees with health problems. Physicians should be especially aware that trust, understanding and showing interest are essential in order for an employee to feel comfortable to disclose person-related information during these conversations. Physicians need to be accessible for employees and need to be aware that time frames are crucial when talking about person-related factors. During the conversation, we recommend that physicians listen to the employee and ask open questions regarding different subjects, such as the employee’s work, thoughts about the future, complaints, and about possible ways to help the employee. This increases the knowledge of the physician about the employee and the employee’s situation and can prove to be beneficial in the development of conversations addressing person-related factors.

This study indicated that—from employees perspective—the most crucial prerequisite for discussing person-related factors during consultations is trust. Therefore, it is important that future research examines how mutual trust between physician and employee can arise, be maintained, or be increased. However, numerous factors were identified which can negatively influence the conversation about person-related factors, making discussing these factors a complex process. This might be one of the reasons why some physicians, according to the participants, do not always ask about all these person-related factors. Future research might be needed to examine the reasons why physicians do not always discuss all person-related factors, or to study the factors that make discussing these factors difficult from the perspective of physicians. Despite the complexity of conversations concerning person-related factors, as far as we know, there is no tool or training available to help OPs and IPs structure these conversations. We recommend that researchers use the information from this study to develop such a tool or training program. Additionally, considering all person-related factors during consultations is time-consuming for the physician. Therefore, it is also of importance that future researchers determine whether considering person-related factors during consultations really improves the practices of OPs and IPs to increase work participation of employees with health problems.
